# Influence of the tubular network on the characteristics of calcium transients in cardiac myocytes

**DOI:** 10.1371/journal.pone.0231056

**Published:** 2020-04-17

**Authors:** Miquel Marchena, Blas Echebarria

**Affiliations:** Departament de Física, Universitat Politècnica de Catalunya, Barcelona, Spain; Cinvestav-IPN, MEXICO

## Abstract

Transverse and axial tubules (TATS) are an essential ingredient of the excitation-contraction machinery that allow the effective coupling of L-type Calcium Channels (LCC) and ryanodine receptors (RyR2). They form a regular network in ventricular cells, while their presence in atrial myocytes is variable regionally and among animal species We have studied the effect of variations in the TAT network using a bidomain computational model of an atrial myocyte with variable density of tubules. At each z-line the t-tubule length is obtained from an exponential distribution, with a given mean penetration length. This gives rise to a distribution of t-tubules in the cell that is characterized by the fractional area (F.A.) occupied by the t-tubules. To obtain consistent results, we average over different realizations of the same mean penetration length. To this, in some simulations we add the effect of a network of axial tubules. Then we study global properties of calcium signaling, as well as regional heterogeneities and local properties of sparks and RyR2 openings. In agreement with recent experiments in detubulated ventricular and atrial cells, we find that detubulation reduces the calcium transient and synchronization in release. However, it does not affect sarcoplasmic reticulum (SR) load, so the decrease in SR calcium release is due to regional differences in Ca^2+^ release, that is restricted to the cell periphery in detubulated cells. Despite the decrease in release, the release gain is larger in detubulated cells, due to recruitment of orphaned RyR2s, i.e, those that are not confronting a cluster of LCCs. This probably provides a safeguard mechanism, allowing physiological values to be maintained upon small changes in the t-tubule density. Finally, we do not find any relevant change in spark properties between tubulated and detubulated cells, suggesting that the differences found in experiments could be due to differential properties of the RyR2s in the membrane and in the t-tubules, not incorporated in the present model. This work will help understand the effect of detubulation, that has been shown to occur in disease conditions such as heart failure (HF) in ventricular cells, or atrial fibrillation (AF) in atrial cells.

## Introduction

Cell contraction in the heart is mediated by a periodic increase of calcium in the cytosol. This is due to the release of calcium stored in the sarcoplasmic reticulum (SR), through specific channels (ryanodine receptors, RyR2) that are sensitive to calcium entering the cell. The RyR2s are distributed spatially in clusters in an almost periodic manner, giving rise to a global release, in a process known as Ca^2+^-induced Ca^2+^ release (CICR). In ventricular cells, the cell membrane has prominent penetrations into the cytosolic space called transversal tubules (t-tubules). The presence of t-tubules implies, directly, a presence of L-type calcium channels (LCCs) in the internal space of the cell and, thus, a simultaneous calcium release when whole cell electrical activation takes place.

The role and presence of t-tubules in atrial myocytes is still not completely understood. Originally, t-tubules were assumed to be a property of ventricular cells, with a very minor presence in atrial myocytes. For instance, confocal microscopy in cat atrial myocytes revealed non-homogeneous spatial calcium profiles caused by the absence of t-tubules [1]. T-tubules were also assumed to be absent in rat atrial myocytes [2], although, nowadays, it has been proven that they present a minor lattice of transversal and axial tubules (TATS) [3]. In particular, the presence of axial tubules in the atria may have an important effect for the activation of the calcium transient [4]. In addition, it was shown that TATS are more relevant in the left atrium, generating higher pressures than in the right atrium. Lately, important improvements in the techniques to analyze TATS have been developed [5, 6], allowing a better determination of the tubular structure.

Although both ventricular and atrial mycoytes may present t-tubules, comparison between them [7] suggests strong differences. In the former, the presence of a highly ordered t-tubule lattice enhances the whole synchronization. In the latter, it is suggested that some myocytes have membrane intubation which accelerate the centripetal wave propagation. Moreover, depending on the mammal, the density of t-tubules varies. In general, the larger the mammal is, the more t-tubules it has [7]. In this respect, it has been shown the prominent presence of t-tubules in cow, horse and sheep, as well as in human cells [8]. For instance, sheep atrial myocytes posses an extensive t-tubule network that synchronizes the Ca^2+^ profile [9], showing a small the peak to peak time delay between the membrane and interior. There exist also differences between right and left atrial myocytes. For example, Arora et al. [10] observed that, in the case of dogs, t-tutubles are more prominent in the left atria than in the right atria. There is also present an inter-cellular variability in the amount of t-tubules from single subjects, probably related to the functional roles of different cell populations [11]. Besides the density of t-tubules, the conformation of these intubations changes during depolarization to produce the heart contraction, with an increase in both volume and length of t-tubules during contraction [12].

In addition, it has been showed that the t-tubule density and structure change during atrial fibrillation (AF) and heart failure (HF). During HF the number of t-tubules is highly reduced. T-tubule remodeling leads the transition between hypertrophy and HF [13]. Differential t-tubule remodeling processes were observed between left and right ventricular myocytes. Persistent AF has also been related to structural remodeling: uncoupling between t-tubules and RyR2 channels; changes in the extracellular matrix [14]. Recent studies highlight a possible role for t-tubule remodeling (disorganization) in dyssynchronization of SR Ca^2+^ release sites in heart failure [15, 16]. In particular, submicron changes are induced due to the transition to HF: Gomez et al. [17] proposed that the gap between LCCs and faced RyR2s enlarges during remodeling (resulting in “orphaned” RyR2s [18]). This has been supported by Meethal et al. [19], who observe that Ca^2+^ sparks are not uniformly distributed within HF cells and disappear from areas devoid of t-tubules, leading to a loss of local control and Ca^2+^ instability in heart failure. T-tubules also affect the current carried by the NCX, which is activated by the release from the RyR2s, mostly located in proximity of the t-tubules [20]. Besides, it has been observed that spontaneous Ca^2+^ sparks do not appear uniformly in the cell, but they occur around the RyR2s close to the tubules [3]. Modeling studies have shown a relation between the density and spatial organization of t-tubules and the occurrence of alternans and triggered activity [21–23].

In this study, we investigate the role of the t-tubular network in terms of calcium wave penetration and homogenization of calcium profiles. We demonstrate that t-tubules promote synchronization in the calcium profiles by studying the calcium levels on the cytosol and the SR, the time delay between internal and peripheral spaces and the peak ratios. In addition, we study the affectation of detubulation on the local coupling dynamics and the RyR2 activity. In particular, we focus on the time to release of an RyR2 and the opening probabilities.

## Materials and methods

We use a modification of a model previously presented in [24]. The main equations that describe the model are:

$$\begin{array}{ccc} \frac{dc_{i}\left(r,t\right)}{dt} & = & J_{i}\left(r,t\right)+\nabla \cdot [D_{i}\left(r\right)\nabla c_{i}\left(r,t\right)]-J_{bi}\left(r,t\right) \end{array}$$ (1)

$$\begin{array}{ccc} \frac{dc_{sr}^{tot}\left(r,t\right)}{dt} & = & \frac{v_{i}\left(r\right)}{v_{sr}\left(r\right)}J_{sr}\left(r,t\right)+\nabla \cdot [D_{sr}\left(r\right)\nabla c_{sr}\left(r,t\right)] \end{array}$$ (2)

$$\begin{array}{ccc} \frac{dc_{bi}\left(r,t\right)}{dt} & = & J_{bi}\left(r,t\right), \end{array}$$ (3)

where *J*_*i*_ and *J*_*sr*_ are the fluxes into the cytosol and the SR spaces, respectively, *J*_*bi*_ accounts for the binding of free calcium to the different buffers. In order to relate the fluxes between the cytoplasm and the SR, we have multiplied the fluxes by the volume fraction *v*_*i*_/*v*_*sr*_, that depends on space, with different values whether the point is close to the z-line or in the inter z-line space. In addition, each point may have different components (RyR2 or not, LCC or not) and may belong to the membrane or not. The fluxes that contribute to the total flux into the cytosol *J*_*i*_ are the SR release flux *J*_*rel*_, the SERCA pump *J*_*up*_, the L-type calcium flux *J*_*CaL*_ and the sodium-calcium exchanger flux *J*_*NaCa*_. All the details of the model can be found in [24]. For this paper, we have incorporated to the model the dynamics of calsequestrin (CSQ), a buffer that affects the calcium on the SR. This buffer is considered to be fast based on their rate constants [25, 26] compared with the release time scale. For that reason, we have applied the rapid buffer approximation to CSQ following [27] (see S1 Data). To apply this approximation, we solve the equations for the total calcium concentration in the SR ($c_{sr}^{tot}$) and then, the free calcium concentration in the SR is calculated using Eq. S5 (see S1 Data). Besides, some parameters are modified with respect to the data published in [24] (see S1 Data for full details on the model).

**Fig 1:**
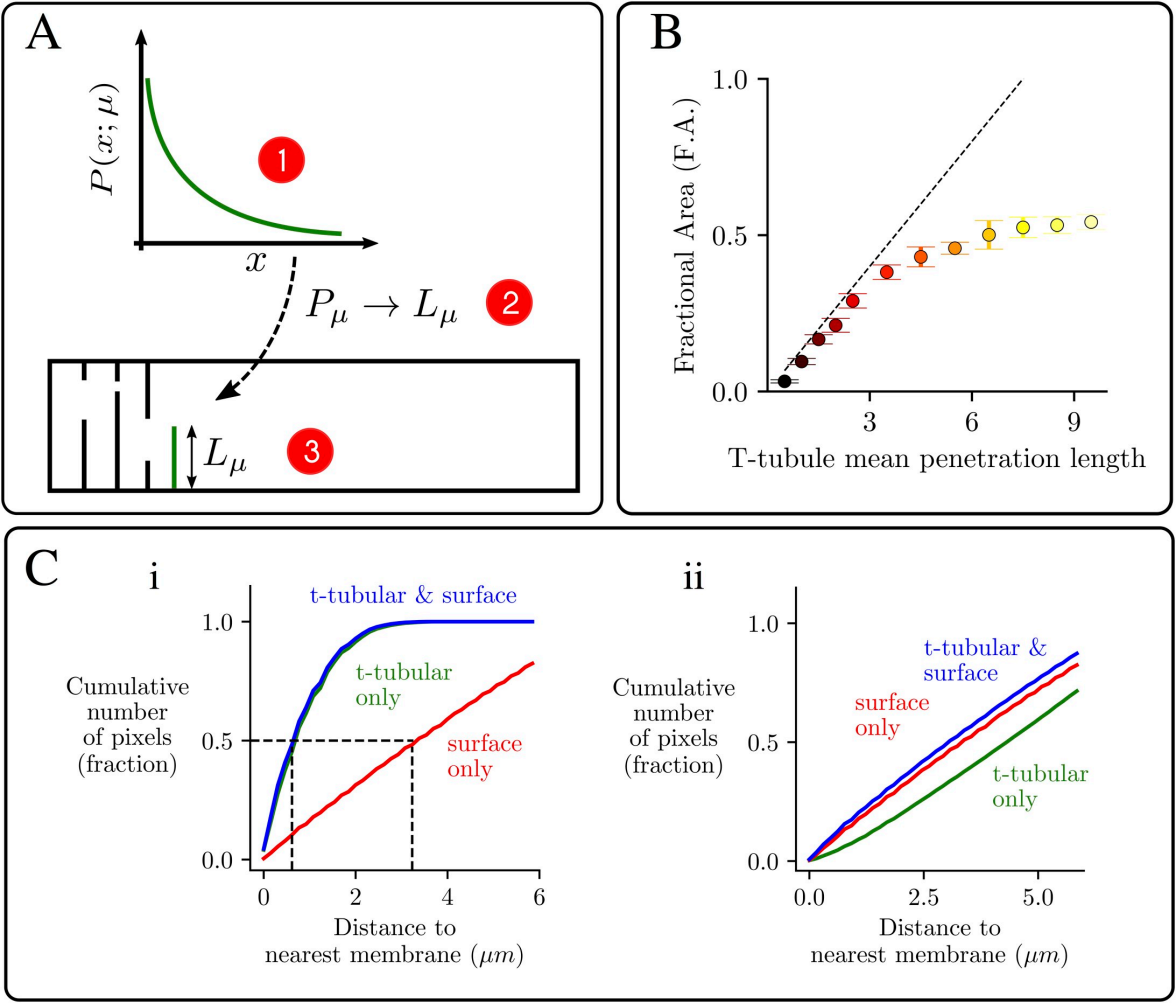
Characteristics of the t-tubule network. A: Schematics of the protocol used for the creation of the t-tubular network in the model. B: Fractional area occupied by the LCCs as a function of *μ*. For each value of *μ* ten different configurations of the t-tubular network have been averaged. The dashed line is the linear function 2*μ*/*L*_*y*_. C: Cumulative number of pixels at a distance from any membrane smaller than a given one, showing contributions of t-tubule and surface membrane. i) Result from the current model for i) F.A. = 0.466 and ii) F.A. = 0.033.

To simulate the effect of the TATS we include points in the interior of the cell that present both the NaCa exchanger pump and LCC channels. For the t-tubules, we consider intubulations of a given length, where we assume that this length (inward penetration) follows an exponential distribution

$$\begin{array}{c} P\left(x;\mu \right)=\frac{1}{\mu }e^{-x/\mu } \end{array}$$ (4)

where *μ* is the mean penetration length of the t-tubules. To generate a random configuration, we follow three steps: 1) pick an exponential random number: *P*_*μ*_, 2) scale this value with the spatial length: *L*_*μ*_ = *L*_*y*_*P*_*μ*_. This random number *L*_*μ*_ is the inward penetration for the first t-tubule. 3) Repeat the process for all t-tubules. These steps are summarized in Fig 1A.

Moreover, we have investigated the axial tubules contribution to the calcium handling. In a similar fashion to the transversal tubules case, we have simulated the existence of these axial tubules by including longitudinal chains that present the NaCa exchanger pump and LCC channels. We assume that the axial tubules length follows a Gaussian distribution, with average length of 1.7*μ*m and standard deviation of 1*μ*m.

### Analysis

Once the t-tubule distribution is set, the internal space of the cell is partially occupied by the LCCs of the t-tubules and associated NCX pump. We can calculate the fractional area (F.A.) occupied by the t-tubules in comparison with the case of fully occupation, i.e., the t-tubules cross through all the transversal section of the cell. To analyze the dependence of the F.A. with *μ* we have simulated ten random configurations for the same value of *μ* following the probability distribution of Eq (4). The mean F.A. is shown in Fig 1B. For low values of *μ* the relation between the F.A. and *μ* follows the theoretical straight dashed line 2*μ*/*L*_*y*_. As *μ* increases, the length of the t-tubules could be greater than *L*_*y*_/2, producing a possible overlap between opposite t-tubules. To avoid this situation, the algorithm limits the length of the t-tubules to *L*_*y*_/2. For that reason, the mean F.A. is smaller than 2*μ*/*L*_*y*_ for large values of *μ*. In the following, we will use F.A. as a measure of t-tubules penetration.

A common measure of t-tubule density is given by how close a given point in the cell is to the nearest t-tubule compared with the cell membrane [8, 9, 28, 29]. This calculation provides us with a quantitative idea of the importance of t-tubules. As an example, we show in Fig 1C data in a control case with the presence of t-tubules (i), and the corresponding data for a cell with a decreased t-tubule density, as for instance, in HF (ii). One can compare those figures with the experimental ones obtained, for instance, in [9], where Fig 1Ci would correspond to the t-tubular network of a sheep ventricular myocyte (Fig 3B in [9]), and Fig 1Cii to that of a sheep atrial myocyte in an animal presenting heart failure (Fig 6B in [9]). The corresponding values of the F.A. (F.A. = 0.466 and F.A. = 0.033, respectively) would be consistent with values measured experimentally, of ∼ 0.3 for ventricular myocytes and up to ∼ 0.1 for sheep atrial myocytes [30]. Typical branch length in atrial myocytes varies from 1.5-2*μ*m in small mammals (rat, mouse, rabbit) to ∼ 2*μ*m in humans [31]. In our model this again would correspond to a F.A. of about 0.1-0.2 (Fig 1), that we can take as our typical value for an atrial myocyte.

## Results

### Calcium transients

**Fig 2:**
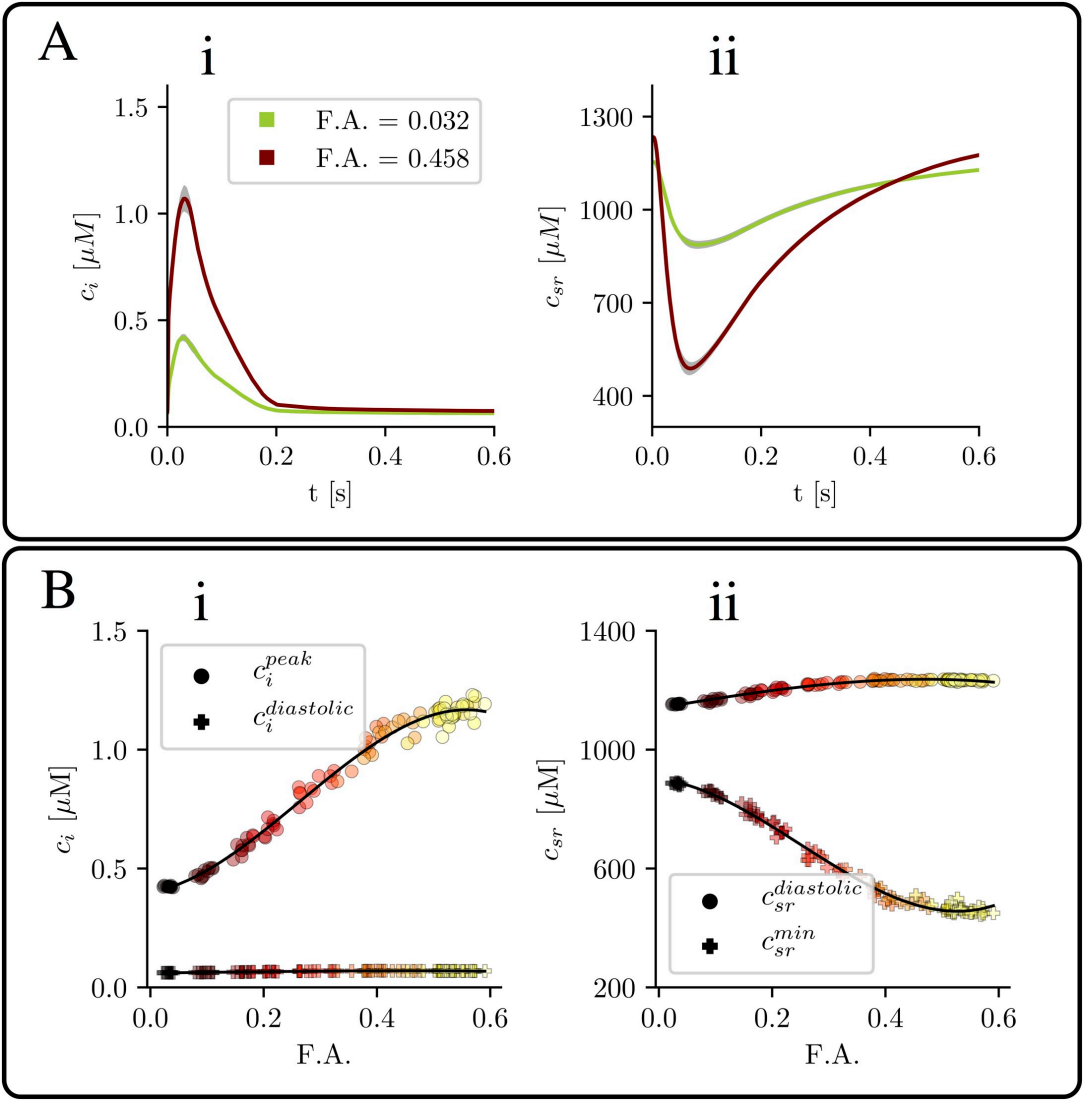
Ca^2+^ transient characteristics as a function of F.A. A: Mean value of calcium concentration (i) in the cytosol and (ii) in the SR, for two different values of the F.A. The dispersion is calculated with ten different configurations of the t-tubular network. B: calcium peak and baseline as function of the F.A. in the cytosol (i) and the SR (ii). Each point represents the mean value over the last 24 beats and one configuration of the t-tubular network. Points with the same color come from the same value of *μ*, the parameter in the length distribution of t-tubules. The solid line is an eye guide to follow the behavior. In all the figures the pacing period is 800 ms.

We first start analyzing properties of the global Ca^2+^ transient. As expected, the average value of the Ca^2+^ concentration is very dependent on the amount of t-tubules (Fig 2). Besides, we observe that, for a given penetration length *μ*, the cytosolic Ca^2+^ peak depends on the specific realization considered (see gray area in Fig 2A). This effect is more visible in the case of a larger t-tubule density. In order to quantify this behavior, we have averaged both peak and diastolic cytosolic calcium values for ten configurations with the same value of *μ* but different values of the F.A. The equivalent calculation has also been done for the SR content. The results are shown in Fig 2B(i) and 2B(ii). In the cytosol (Fig 2Bi) the diastolic value does not depend on the F.A. However, the peak value highly increases with the amount of t-tubules, with a more than 2-fold increase. A similar trend is observed in the case of the SR content (Fig 2Bii), where the minimum value decreases with the F.A., while the diastolic value remains almost constant. Thus, the increase in fractional release is not related to a higher SR load. We will later study in more detail this effect.

**Fig 3:**
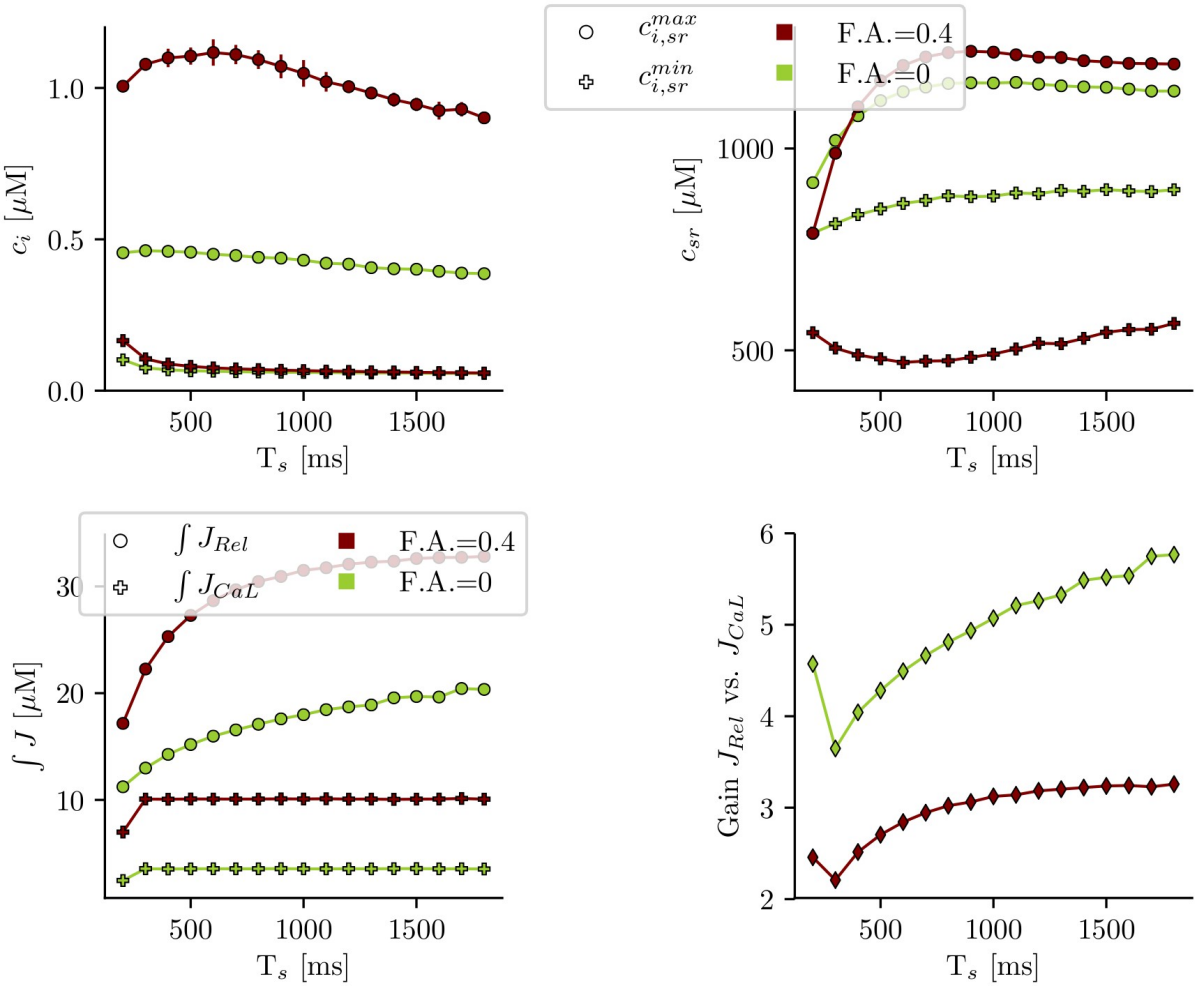
Dependence on the pacing period. Top: calcium peak for different values of the pacing period (T_*s*_) for two scenarios: the loss of t-tubules due to HF (green) and the control situation with t-tubules (red). Bottom: average Ca^2+^ release and influx (in *μM*), obtained integrating the fluxes *J*_*rel*_ and *J*_*CaL*_ over a period and averaging over 20 stimulations (same color coding).

The disruption of the t-tubular system may have a considerable impact in the regulation of atrial contraction [9]. It is, for instance, possible that the cell has a different response to fast pacing frequencies. In Fig 3 (top) we show the calcium peak and SR load dependence with the pacing period for the cases of a small and large t-tubule density. In both cases, the diastolic value of SR load and the cytosolic Ca peak increase slightly first with pacing frequency, and then decrease at frequencies larger than about 2Hz. There is also a continuous decrease of the release flux with frequency, that becomes more abrupt at larger frequencies, and it is probably due to refractoriness of release [32, 33]. Both the release flux and the calcium influx through the LCC are smaller in the case of disrupted t-tubules, although the decrease in the former is not as pronounced as in the latter, due to a larger EC gain in detubulated cells, thus partly avoiding the effects of the reduced calcium influx. From this, it arises the idea that the dynamics is robust enough to maintain always physiological values.

**Fig 4:**
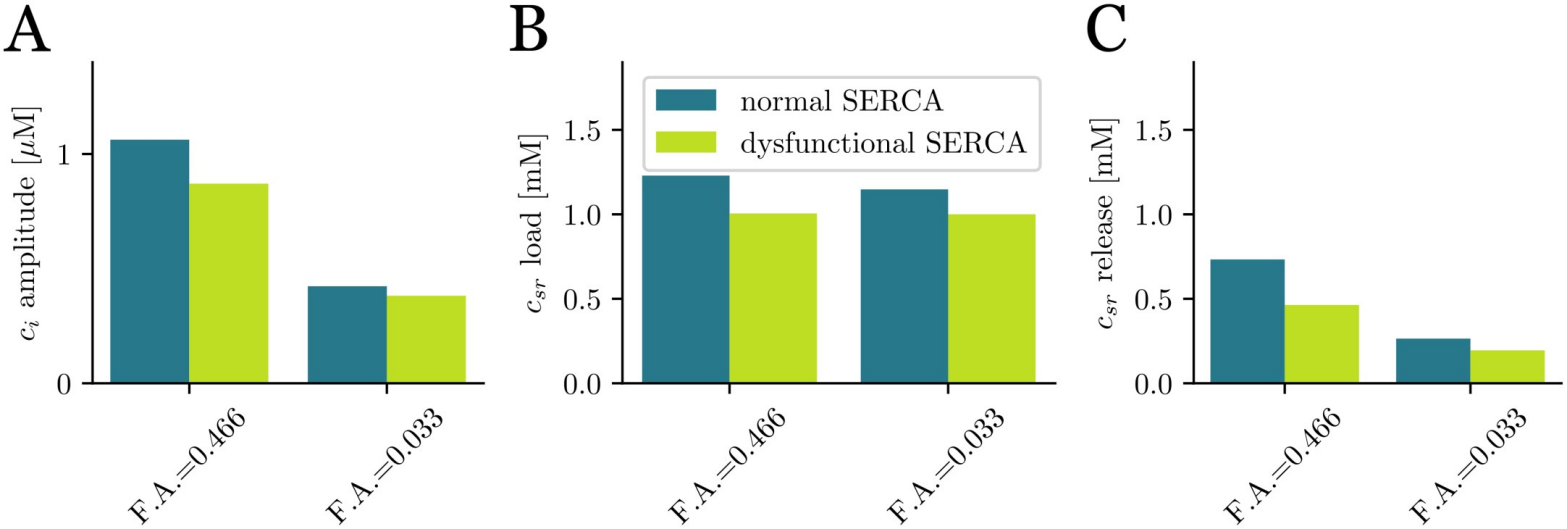
Effect of a dysfunctional SERCA pump. Values of A: cytosolic Ca^2+^ peak; B: SR Ca^2+^ load; C: SR fractional release, for the control case and reduced activity of SERCA. The pacing period is 800 ms.

Often, t-tubule disruption is accompanied by a decrease in SERCA strength, as, for instance, during HF [34, 35]. We have checked the effect of this dysfunction by modifying two parameters of the SERCA pump: the strength of SERCA, *g*_*up*_, has been decreased 30%, while its cytosolic calcium dissociation constant, *K*_*i*_, has been increased by 30%. This results in a slower SERCA, with lower affinity for cytosolic calcium, producing a reduction of about a 17-18% in the calcium transient and SR Ca^2+^ load (Fig 4), in the case of tubulated cells (F.A. = 0.466), and a slightly lower reduction of a 9-13% for detubulated cells (F.A. = 0.033). As also happened in the control case (see Fig 2), for the modified SERCA function, SR Ca^2+^ load does not change much with the amount of t-tubules, while both the fractional release and the amplitude of the calcium transient are reduced in about a factor 2 when the fractional area is reduced from F.A = 0.466 to F.A. = 0.033.

### Inward propagation

**Fig 5:**
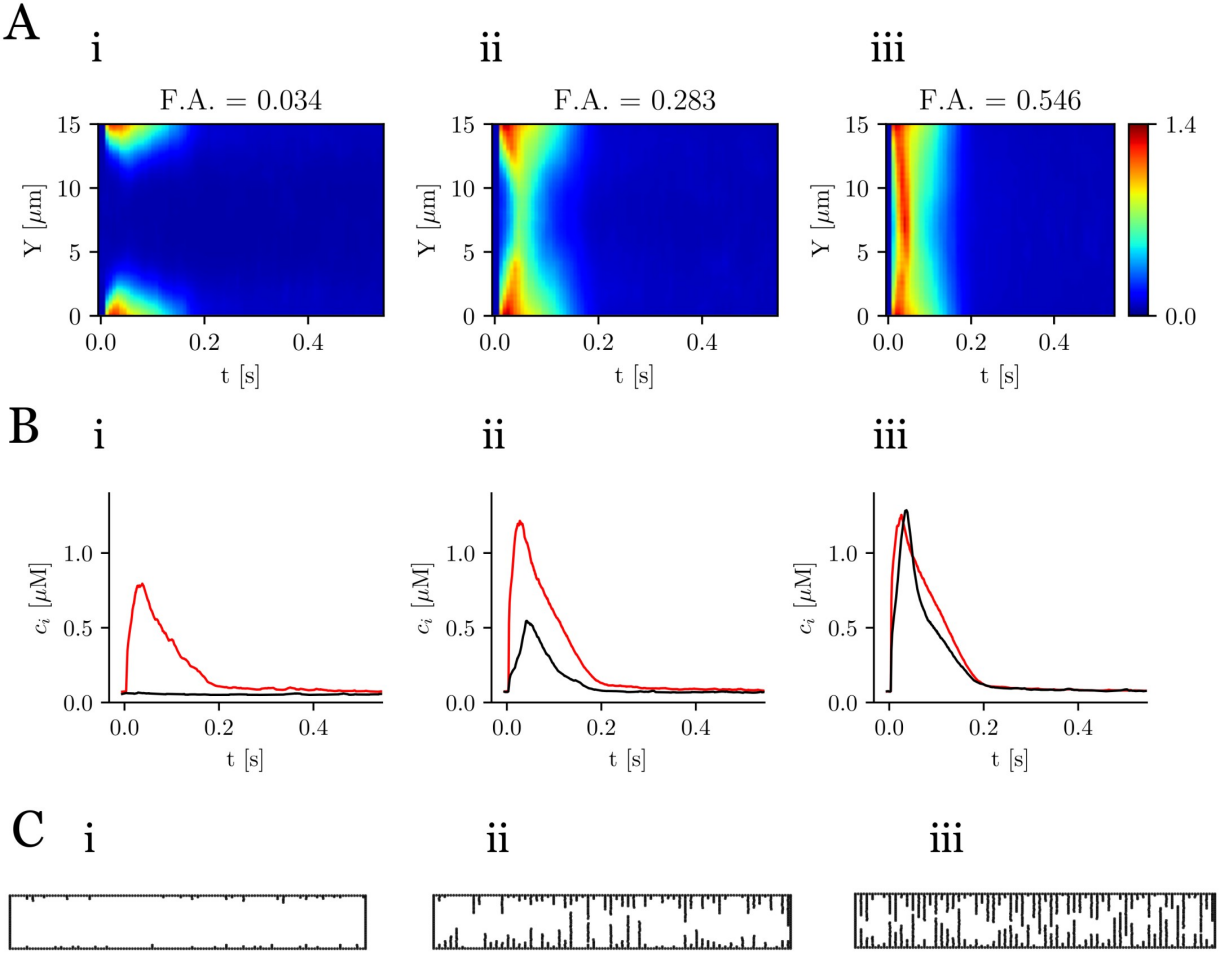
Spatial Ca^2+^ profiles. A: line scans averaged over the longitudinal direction for three values of the F.A. The colorbar corresponds to calcium concentration in *μ*M. B: mean value of calcium close to the membrane (red) and in the center of the cell (black). C: the three t-tubular network configurations. Pacing period of 800 ms.

The presence of t-tubules in atrial cells enhances the homogenization of calcium profiles, i.e. the synchronization between the center of the cell and peripheral region. Inward calcium propagation is highly dependent on the t-tubules distribution. This can be observed plotting the averaged calcium content in two regions: close to the membrane (red line in Fig 5) and in the central region (black line in Fig 5). The average line scan is calculated using:

$$\begin{array}{c} {\left\langle c\left(x,y\right)\right\rangle }_{x}=\int_{0}^{L_{x}}c\left(x,y\right)dx, \end{array}$$ (5)

where *c*(*x*, *y*) is the calcium concentration at (*x*, *y*). For low values of F.A. the peak in the center is practically nonexistent (Fig 5Bi). This contrasts with the prominent peak in the internal region for high values of F.A. This increment on the calcium content in the cytosol is produced by the increase of the fractional release in the SR, as we have already pointed in Fig 2D. Thus, there is a strong correlation between the t-tubular geometry (shown in Fig 5C) and the calcium dynamics.

**Fig 6:**
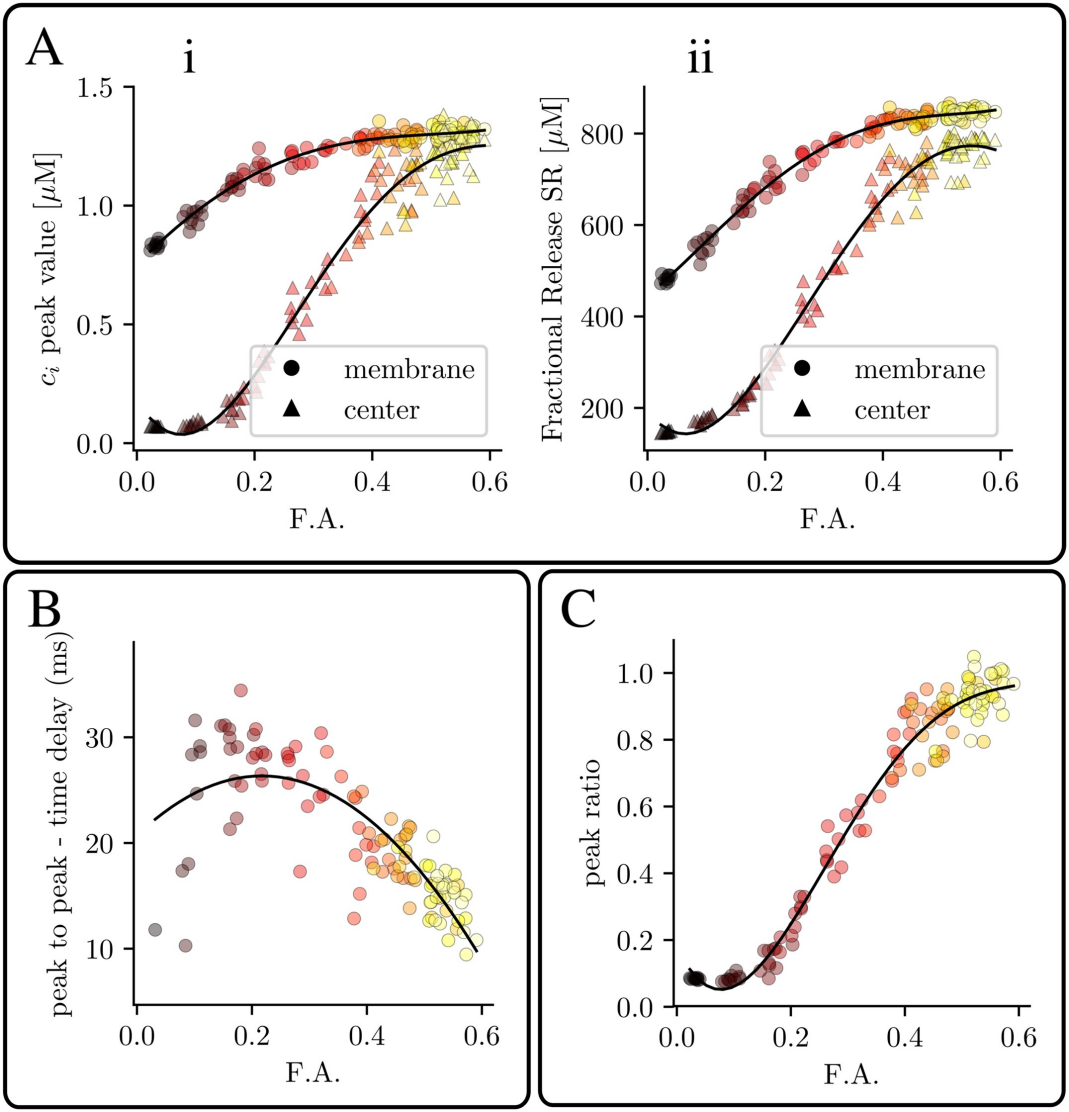
Inhomogeneity in release as a function of t-tubule density. Ai: cytosolic calcium peak close to the membrane and in the center of the cell as function of the F.A. Aii: fractional release from the SR close to the membrane and in the center as function of the F.A. B: delay time between peripheral and central regions as function of the F.A. C: Peak ratio between peripheral and central region ($Ca_{ct}^{2+}/Ca_{ss}^{2+}$) as function of the F.A. Pacing period of 800 ms.

Related to this, we have calculated the cytosolic calcium peak (Fig 6Ai) and SR fractional release (Fig 6Aii) close to the membrane and in the central region for several values of the F.A. Fig 6A show that full inward propagation is only achieved for the highest value of the F.A. In addition, we show the delay time between subsarcolemmal and internal peaks in Fig 6B. For low values of the F.A., the central peak is small and, in many cases, almost nonexistent, so that the delay time, if present, is high. As the F.A. increases both peaks become more synchronized meaning that the internal space is triggered simultaneously with the submembrane. This synchronization is also observed in the ratio between subsarcolemmal and central peaks. Fig 6C shows that for low values of the F.A., this ratio tends to zero, given that the peak in the internal region tends to vanish. On the other limit, for high values of the F.A., both peaks become synchronized and this ratio tends to 1.

#### Effect of axial tubules in calcium propagation

**Fig 7:**
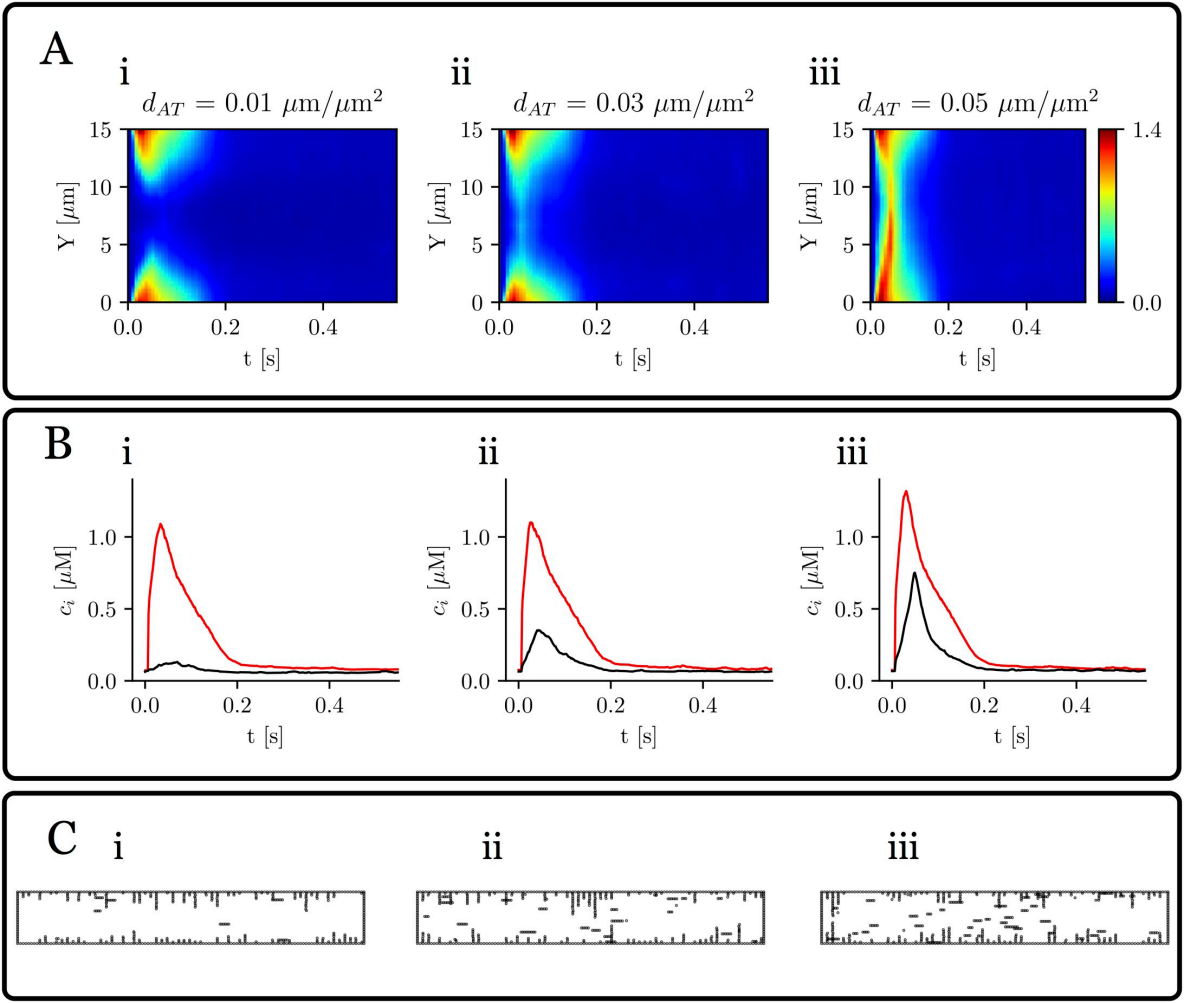
Effect of axial tubules. Calcium traces as a function of axial tubule density *d*_*T*_, defined as the ratio of axial tubules total length and cell surface. A: line scans averaged over the longitudinal direction for three values of *d*_*T*_ = 0.01, 0.03, and 0.05 *μ*m/*μ*m^2^, for a fractional area of t-tubules of F.A. = 0.16. The colorbar corresponds to calcium concentration in *μ*M. B: mean value of calcium close to the membrane (red) and in the center of the cell (black). C: the three tubular network configurations. Pacing period of 800 ms.

To study how dependent calcium inward propagation is on the amount, not only of t-tubules, but also of axial tubules, we have redone some of the simulations, including a varying density of axial tubules. In Fig 7 we show the results, for a given value of the F.A. of t-tubules that in the absence of axial tubules results in failure of inward propagation. We observe that, increasing the density of axial tubules, a peak in cytosolic calcium in the central region is obtained, with a delay with respect to the peak at the subsarcolemmal region. In the linescans, this results in a V shape (Fig 7), as has been often described experimentally [28].

### Local dynamics

In the previous sections we have observed that, as the amount of t-tubules decreases, there is a delay between the rise of calcium close to the membrane and in the interior of the cell. This spatial dyssynchronization can be studied in more detail computing the release characteristics of individual RyR2s in the cell. In this analysis, we have recorded the open time for all the RyR2s during 19 beats and different values of the F.A.

**Fig 8:**
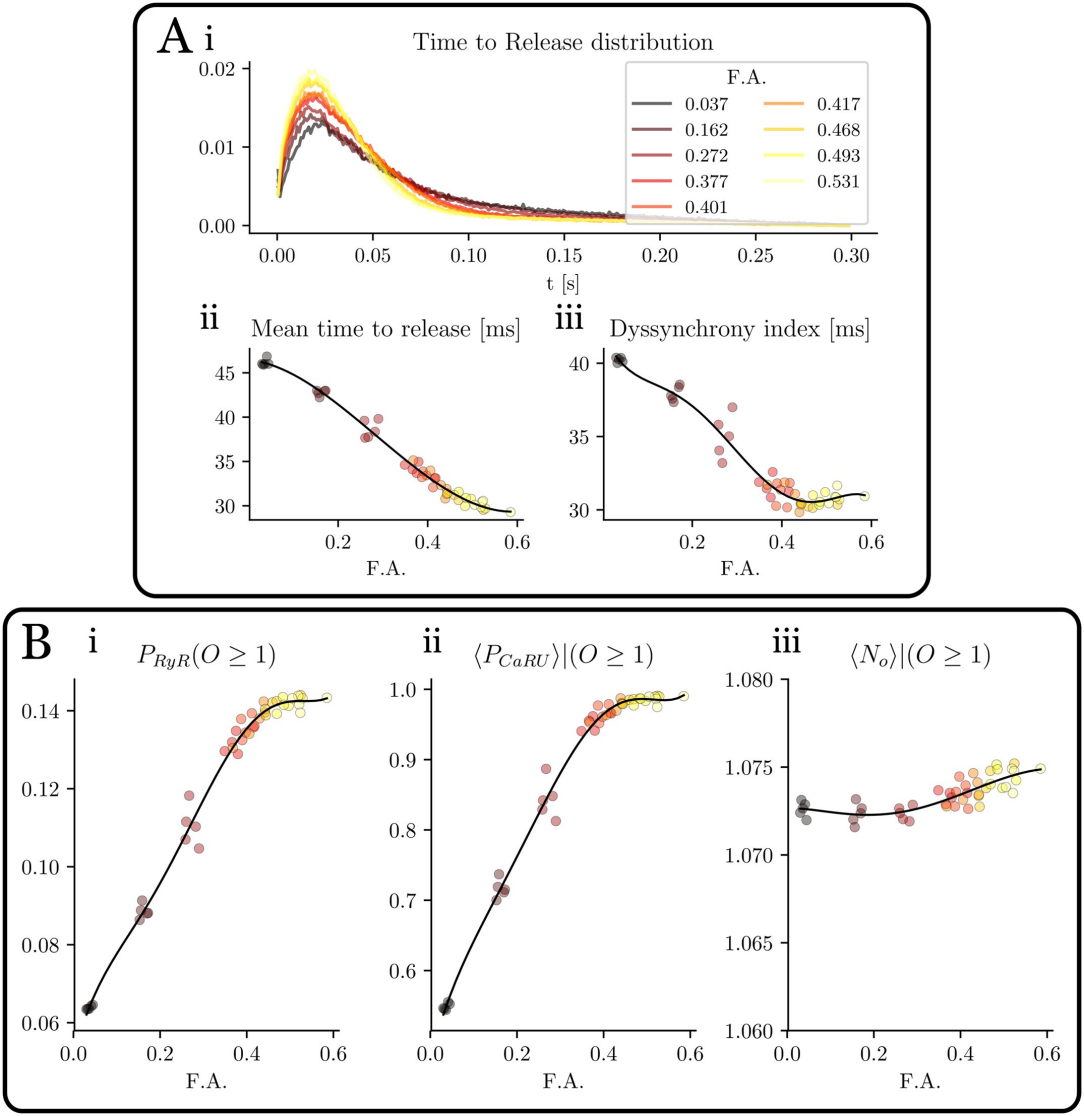
Statistical measures of RyR2 activity. A: time to release distribution (i), average time to release (ii) and dyssynchrony index (iii) as function of the F.A. B: Dependence on the F.A. of (i) fraction of RyR2 that open, (ii) fraction of CaRU with at least one RyR2 that opens and (iii) average number of times a given RyR2 opens in a given beat. Pacing period of 800 ms.

The time to release (*t*_*tr*_) is defined as the time passed between the beginning of the stimulation and the first release of a RyR2. This time depends on many factors: diffusion coefficient, SR load, opening rate in the RyR2 stochastic model, etc. In particular, simulations show that *t*_*tr*_ heavily depends on the t-tubule density. In Fig 8Ai the probability distribution of *t*_*tr*_ is shown for different values of the F.A. The bigger the F.A., the higher and narrower the peak becomes. Also the mean value of *t*_*tr*_ decreases with the F.A. (Fig 8Aii). Once a given RyR2 has opened, it typically does not open again during the same stimulation, i.e, the number of times each individual RyR2 opens (Fig 8Biii) is approximately always one and it does not change with F.A. Once a RyR2 opens it remains on the open state an average time of around 12-13ms, that is independent of the t-tubular density (see S1 Data).

A usual characterization of RyR2 release synchronization is obtained with the Dyssynchrony Index (DI), defined as the standard deviation of the *t*_*tr*_ distribution. This clearly shows that an increase of t-tubule density enhances RyR2s synchronization (Fig 8Aiii). It is not just that RyR2 release is more synchronized, but, as the t-tubule density increases, the ratio of RyR2s that open also increases. We have defined the opening probability (*P*_*o*_) of the RyR2s as the number of RyR2s that have opened at least once (*N*_*RyR*_(*O* ≥ 1)) in a given beat, divided by the total number of RyR2s *N*_*RyR*_. It increases almost linearly with t-tubule density, up to a point where it saturates. Interestingly, if we compute, not the individual RyR2s, but the fraction of CaRUs where, at least one RyR2 opens, we see that it reaches almost the value one, for a F.A. of 0.5. Thus, at this point, all CaRUs fire, some by direct stimulation from the LCCs at the t-tubules, others, due to recruitment from adjacent CaRUs.

Finally, we have studied the frequency and characteristics of sparks, under rest conditions (without an external stimulation). In this case, we do not observe any significant change in the distribution and properties of sparks as a function of F.A. (Figs 3,4 in S1 Data).

## Discussion

The TAT network has long been recognized as an essential ingredient to ensure excitation-contraction coupling and subcellular synchronized Ca^2+^ release. Contrary to ventricular cells, that present a well organized t-tubular network, the amount of t-tubules in atrial cells depends on the animal species [7, 8] and within a given species, on the location of the cell in the atria [10, 11]. When present, t-tubules help to synchronize the rise of the systolic Ca^2+^ transient also in atrial cells [9, 14]. Probably linked to this, the central calcium transient has been found of similar amplitude than the initial subsarcolemmal transient in some species [1], while in others it is significantly reduced [36]. T-tubules are often disrupted in diseased conditions, such as heart failure in the ventricles [13, 16, 18, 37] or atrial fibrillation [14]. In both cases, the loss of t-tubule structure results in a decreased calcium transient and loss of synchrony in release.

Previous modeling studies have addressed the relation between t-tubule density and spatial organization, and the appearance of alternans and triggered arrhythmias. Song et al [22], in a model of a ventricular myocyte, found that alternans were promoted at intermediate values of the t-tubule density, when the fraction of orphaned RyR2s was in an intermediate range. In this regime, the LCC clusters and NCX are properly matched so that the CaRUs can be sufficiently synchronized by the LCC clusters and spontaneous calcium releases are not suppressed by NCX, potentiating triggered arrhythmias. T-tubule disruption was also found to give rise to a steeper SR Ca release-load relationship, predisposing for the appearance of alternans. For atrial myocytes, on the contrary, Colman et al [23] found that alternans appears at low values of t-tubule density, since this results in alternating behavior of success and failure of inward calcium wave propagation. Spontaneous Ca waves originate from regions with no T-tubules, probably because the lack of NCX allows a local spontaneous release to trigger neighboring RyR2 clusters.

In this paper we have studied the effect of modifying the amount of t-tubules in a subcellular model of a cardiac cell [24]. The t-tubular structure is reproduced considering transverse t-tubules of variable penetration length, taken from an exponential distribution (Fig 1A), that results in a given t-tubular structure. We have then considered averages over different realizations with the same mean penetration length. Since different realizations could give rise to a different value of the F.A. occupied by t-tubules, we found more useful to use this last variable to characterize the t-tubular network. Interestingly, the network so constructed gives a good agreement with statistical measures of distances to the membrane, obtained experimentally [8, 9, 28].

### Detubulation reduces the Ca^2+^ transient but does not affect SR load

In detubulated ventricular cells, the amplitude of the systolic Ca^2+^ transient and its rate of rise have been observed to be reduced [38, 39], while the rate of decay remains largely unchanged. This is consistent with our results (Fig 2), where the calcium transient increases almost three-fold from purely detubulated cells to cells with a dense t-tubular network. The SR Ca^2+^ load, however, remains almost constant. This agrees with results obtained in detubulated myocytes [39], as well as in during AF, where a decrease in release was observed despite similar SR content [14].

In our simulations, a reduction in the length of t-tubules results in an effective reduction of ICaL and NCX currents (Fig 3). However, both are modified equally so, if they are in equilibrium for a cell with a dense t-tubular network, they are expected to be in equilibrium in a detubulated cell. In terms of Ca^2+^ homeostasis, this means that one expects diastolic values of cytosolic and SR Ca^2+^ to remain almost unchanged. One would expect a similar results if in a tubulated cell one decreases the strength of both ICaL and NCX. This analogy is true except for spatial effects, that may become important at fast pacing rates, where diffusion is not fast enough to equilibrate concentrations, and diastolic values in both cases start to diverge (Fig 3).

### Detubulation and SERCA dysfunction impair release in different manners

In HF, a reduction in the t-tubular network is accompanied by a decrease in SERCA functionality [34, 35]. Although both result in decreased Ca^2+^ transients (Fig 4), their effect is very different. Detubulation reduces release by reducing the amount of CaRUs that fire (Fig 6), thus modifying systolic values of Ca^2+^ (cytosolic Ca^2+^ peak, SR fractional release), but with little effect on diastolic values. It thus directly affects SR release. A reduction in SERCA, on the other hand, results in a change in diastolic Ca^2+^ levels, i.e., a reduction of SR Ca^2+^ load, that produces a reduction of SR release.

### Detubulation produces dyssynchronization in release

Contrary to normal ventricular cells, in atrial and ventricular detubulated cells, a delay is observed between the Ca^2+^ rise close to the membrane and at the cell center [1, 7, 11, 40, 41]. In line scans, this gives rise to a specific U-shaped pattern [7, 11, 41]. This effect is clearly observed in the simulations as the t-tubular F.A. is varied (Fig 4). When the F.A. is reduced, the amplitude of the interior Ca^2+^ transient is much reduced with respect to that close to the cell membrane (Fig 5), while the time delay between both peaks increases.

This dyssynchrony in release can be observed in the statistics of release events at individual RyR2s (Fig 6). A computation of the time at which each individual RyR2 opens for the first time shows that RyR2s start to open upon the entrance of Ca^2+^ into the cell, with a mean time to release that increases in detubulated cells. In this case, the distribution is also broader, as orphaned RyR2s start to open. Defining a dyssynchrony index as the standard deviation of that distribution, one can observe that it increases in detubulated cells. This had already been shown in a computational study [42], using a simpler model for release that accounted for CaRU recruitment, but with instantaneous opening of a fraction of the RyR2s.

Interestingly, the number of CaRUs that open at least once increases with F.A. in an almost linear fashion (Fig 6), but is always larger than the fractional area, meaning that many orphan RyR2s open. Even in the total absence of t-tubules, about half of the CaRUs open (Fig 6).

### Axial tubules enhance inward calcium propagation

Besides transverse tubules, axial tubules have been observed in several animal species [4, 31] and, in fact, in some species they can represent the main component of the TAT network [31]. We find that axial tubules have a profound impact for centripetal wave propagation. In situations where the t-tubular network alone does not produce inward propagation in our simulations, a small amount of axial tubules is enough to produce release in the central region (Fig 7). In the absence of axial tubules, for a typical F.A. = 0.1-0.2 of an atrial myocyte our results do not show inward propagation, less alone if it is detubulated, contrary to some experimental observations [38, 43], where detubulation results in a smaller global transient and a delay between the subsarcolemmal and central calcium peaks, but with still an appreciable central calcium transient. This would agree with the presence of axial t-tubules, that reproduce the typical V shape in the linescans obtained in experiments. Interestingly, the density of axial tubules measured in experiments [31] (*d*_*T*_ ∼ 0.04−0.08*μ*m/*μ*m^2^, for small mammals) agrees well with our result that show that the central peak is recovered for that density of axial tubules (Fig 7).

### Orphaned RyR2s increase LCC-release gain in detubulated cells

In detubulated cells there is a decrease in calcium entrance through the LCC. This is partly compensated by an increase in the excitation contraction (ECC) gain function, measured as Δ[*Ca*^2+^]_*rel*_/Δ[*Ca*^2+^]_*influx*_, i.e., total calcium released from the SR compared to total Ca^2+^ entry through the LCC channels (Fig 3). So detubulation results in a reduction of the SR release and cytosolic transient in a less pronounced manner than that of LCC total current. The reason is linked to recruitment of orphaned RyR2s, so the increase in cytosolic Ca^2+^ due to the opening of CaRUs where LCCs are present produces secondary openings of orphaned RyR2s. Thus, release in this case is a mixture of coupled and propagated Ca^2+^ release. This may serve as an adaptive mechanism when t-tubule remodeling occurs [44]. However, it seems to be in contradiction to what has been observed in cardiomyocytes in post myocardial infarction (PMI) animals [45], suggesting that probably other mechanisms beyond detubulation are in play during PMI, i.e., a mismatch or an increased gap between LCCs and RyR2s.

### Above a certain value of the t-tubular density, complete coupling is obtained

The different measures of the Ca^2+^ transient (Ca^2+^ release and peak systolic Ca^2+^, ratio of peripheral to interior Ca^2+^ peak, etc) saturate above a certain density of t-tubules. So, for a F.A. above 50%, the addition of t-tubules does not have any effect on the transients. As we already discussed, this is due to the recruitment of orphaned RyR2, so, for F.A. above around 50%, all CaRUs open (Fig 6B). Interestingly, a value of F.A. close to 50% was measured experimentally in rat ventricular cells [46], that was reduced 2-fold in case of HF.

### Spark properties do not change with detubulation

In detubulated cells, spontaneous sparks have been found close to the cell membrane [3, 41]. This is in sharp contrast with our results, where both the position and characteristics of sparks are the same in tubulated and detubulated cells (Fig 5 in S1 Data). In fact, several possibilities could account for this discrepancy. One is that most sparks appear due to random LCC openings, that trigger the opening of the adjacent RyR2s. Besides, it could be due to local variations of cytosolic Ca^2+^ regulation, due to the presence of NCX at the t-tubules. Higher Ca^2+^ levels at the regions without t-tubules would result in higher RyR2 activity [19]. This is, however, not what we observe in our simulations. This seems to strengthen the possibility of a different regulation the of RyR2s at the t-tubules [3, 47].

#### Limitations

The present study presents several limitations. We present an idealized description of the t-tubular network. For instance, besides transversal, axial tubules have also been found to contribute to rapid activation of the atrial cell [4], and these have not been considered in the model. Thus, the exact details of the t-tubular network deviates from what is found in experiments. Nevertheless, the way we have introduced t-tubules allows for an easy change of their density and still agrees qualitatively well with the average statistical properties found in cells [8, 9, 28, 29]. Another important effect, not included in our model, is the presence of mitochondria. In ventricular myocytes, there is evidence suggesting that the mitochondrial outer membrane is linked to t-tubules [48]. Besides, there maybe regional differences of ICaL function and LCC-RyR2 coupling between the t-tubules and the cell membrane [49], that have not been incorporated into the model. Detubulation in HF or AF is often associated with other physiological changes, as RyR2 sensitivity, changes in transmembrane currents, etc [50]. This may have an important effect in the calcium transient and is not taken into account in this study. Moreover, detubulation in heart failure may be accompanied by AP propagation failure across the TAT network, resulting in asynchronous calcium release and, when present intermittently, local calcium alternans [51, 52]. In our model, a permanent failure of AP propagation would be similar to an effective reduction of the TAT network F.A., with the corresponding decrease in inward propagation and increase in dyssynchrony. As a future work, it could be interesting to consider the case of intermittent AP propagation and how this is related to the occurrence of global calcium alternans.

Our results focus on the effect of detubulation in an otherwise healthy cell (except for the modification in SERCA). Thus, it not a good model to study HF or atrial fibrillation, where other physiological changes occur, but it can help understand which is the contribution of detubulation to the changes observed in remodeled cells. In fact, detubulation could arguably have a different effect in ventricular and atrial cells, due to different physiological conditions of both cells, which we are not taken into account.

In this work we consider a fixed cell size. However, the amount of TAT is typically dependent on cell size [31], even within the same animal species, and it has been observed, in modeling studies, to modulate the effect of t-tubule disruption [22, 23]. Finally, we have considered a voltage clamped cell and therefore do not allow for calcium-voltage coupling. The interplay between detubulation and AP form and duration (with its impact in calcium homeostasis) is a very interesting point for future work.

## Conclusion

In this work we have studied the effect of the t-tubule network on a computational model of an atrial myocyte. As already known from experiments and other modeling studies, we found that detubulation decreases the cytosolic Ca^2+^ transients and increases release dyssynchronization. We trace this last effect to a broadening of the RyR2 release time distribution, due to the late opening of orphaned RyR2. Despite its effect of systolic cytosolic Ca^2+^, detubulation does not produce any significant change in diastolic values, with SR load almost the same. Contrary to experimental observations, we do not find any significant different in spark properties and location of tubulated and detubulated cells, suggesting that maybe another mechanism, not included in our model, is at play in, for instance, HF.

## Supporting information

S1 Data (PDF)
