# Supplementary material for: Influence of the tubular network on the characteristics of calcium transients in cardiac myocytes
Source: PLoS One. 2020 Apr 17;15(4):e0231056. doi: 10.1371/journal.pone.0231056 (PMC7164608; doi:10.1371/journal.pone.0231056)
Supplement: S1 Data — (PDF) [file pone.0231056.s001.pdf]

Influence of t-tubular network on the characteristics of calcium transients in cardiac myocytes
Supporting Information

Miquel Marchena and Blas Echebarria

August 14, 2019

1 Methods

1.1 Calsequestrin dynamics

We have incorporated the effects of CSQ in the model presented in [2]. Calsequestrin is the leading buffer in the SR [1] and is modeled as a linear buffer:

$$[\mathrm{Ca}^{2+}] + [\mathrm{CSQ}] \underset{k_-}{\overset{k_+}{\rightleftharpoons}} [\mathrm{Ca}^{2+}\,\mathrm{CSQ}],$$

where k_+ and k_- are the association and dissociation rate constants. The ordinary differential equation that governs this reaction is:

$$\frac{d[\mathrm{Ca}^{2+}\mathrm{CSQ}]}{dt} = k_+[\mathrm{Ca}^{2+}][\mathrm{CSQ}] - k_-[\mathrm{Ca}^{2+}\mathrm{CSQ}]. \tag{1}$$

This process is considered to be faster than diffusion in the SR and the release time scales. Thus, we apply the rapid buffer approximation:

$$\frac{d[\mathrm{Ca}^{2+}\mathrm{CSQ}]}{dt} = 0 = k_+[\mathrm{Ca}^{2+}][\mathrm{CSQ}] - k_-[\mathrm{Ca}^{2+}\mathrm{CSQ}]. \tag{2}$$

The total concentration of Calsequestrin is: $[\mathrm{B}]_{CSQ} = [\mathrm{CSQ}] + [\mathrm{Ca}^{2+}\mathrm{CSQ}]$. Combining both equations, the total concentration of free Calsequestrin is:

$$[\mathrm{CSQ}] = \frac{[\mathrm{B}]_{CSQ} k_{d,CSQ}}{k_{d,CSQ} + [\mathrm{Ca}^{2+}]}, \tag{3}$$

where $k_{d,CSQ} = k_-/k_+$ is the dissociation constant of the reaction. The total concentration of calcium in the SR is:

$$\begin{aligned} \left[\mathrm{Ca}^{2+}\right]_{Tot} &= [\mathrm{Ca}^{2+}] + [\mathrm{Ca}^{2+}\mathrm{CSQ}] = [\mathrm{Ca}^{2+}] + [\mathrm{B}]_{CSQ} - [\mathrm{CSQ}] \\ \left[\mathrm{Ca}^{2+}\right]_{Tot} &= [\mathrm{Ca}^{2+}]\left(1 + \frac{[\mathrm{B}]_{CSQ}}{k_{d,CSQ} + [\mathrm{Ca}^{2+}]}\right). \end{aligned} \tag{4}$$

Inversely, solving for $[\mathrm{Ca}^{2+}]$ we obtain:

$$[\mathrm{Ca}^{2+}] = \frac{1}{2}\left(-B + \sqrt{B^2 + C}\right) \tag{5}$$

where

$$\begin{aligned} B &= k_{d,CSQ} + [\mathrm{B}]_{CSQ} - \left[\mathrm{Ca}^{2+}\right]_{Tot} \\ C &= 4\left[\mathrm{Ca}^{2+}\right]_{Tot} k_{d,CSQ} \end{aligned} \tag{6}$$

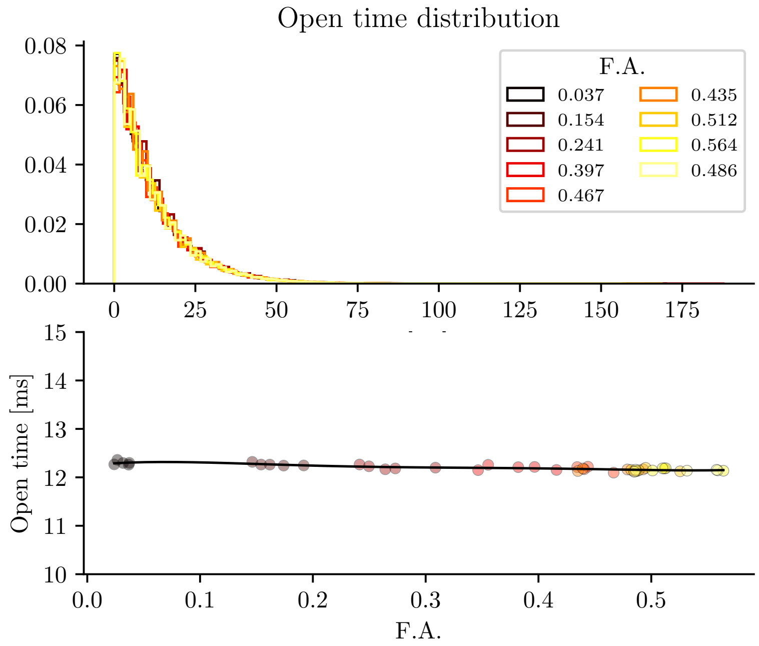

Figure 1: Open time distribution (top) and average open time for different values of the F.A. (bottom), in an stimulated myocyte.

1.2 Set of parameters

The parameters of the model can be found in Tables 1 and 2. Besides the addition of the buffer calsequestrin, three parameters have been adjusted with respect to parameters published on [2]: $g_{NaCa} = 172$ μM/ms (old 54 μM/ms), $k_a = 2.1 \cdot 10^{-3}$ $\mu\text{M}^{-2}\text{ms}^{-1}$ (old $1.77 \cdot 10^{-4}$ $\mu\text{M}^{-2}\text{ms}^{-1}$), and $EC_{50-SR} = 450$ μM (old 350 μM).

2 Results

2.1 RyR2 activity in stimulated cells

The open time (t_o) is defined as the time that a RyR2 is in the open state. In this analysis, we have recorded the open time for all the RyR2s during 17 beats and for a wide range of the F.A. The probability distributions of t_o obtained from the simulations are shown in Fig. 1 (top). The mean values of these distributions are shown in Fig. 1 (down). The overlapping of all distributions and the lack of variation in the mean value suggest that t_o does not depend on the density of t-tubules.

2.2 RyR2 activity under rest conditions

Under rest conditions (with the membrane potential fixed to -80 mV) the open time (t_o) does not change with the fractional area (Fig. 2). It is reasonable to obtain the same result as in the stimulated simulations since the rate between the close state and the open state (k_{oc}) does not depend neither the local concentration of calcium nor the membrane potential. The time

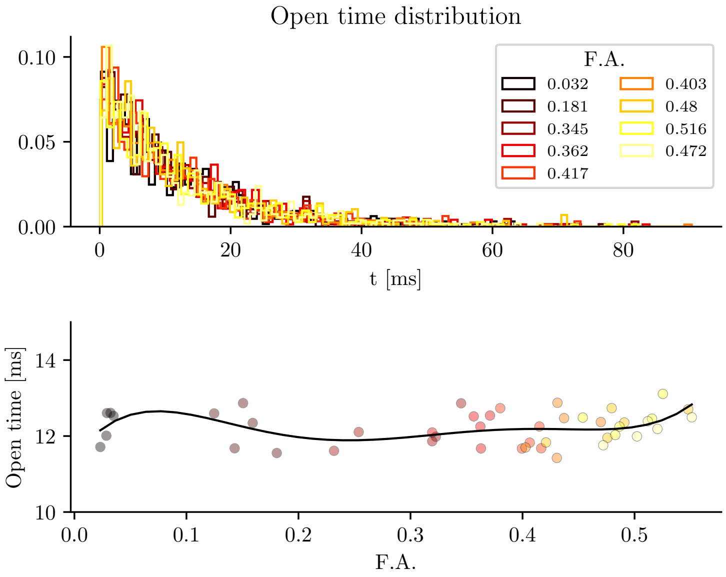

Figure 2: Open time (t_o) distribution (top) and average open time for different values of the F.A. (bottom), under rest conditions. Total time of simulation: 13.5s.

to release distribution shown in Fig. 3Ai is almost flat with a certain randomness. Simulations suggest that the synchronization is produced by the external potential and when it is removed the mean time to release and the dyssynchrony index (Fig. 3A ii and iii) are constant. For that time of simulation, we have around 50 openings per second and the RyR2s open at most one time.

2.3 Spark properties

In Figs. 4 and 5 we show the spark properties for different values of the F.A. We have measured different features of the sparks: the time when the RyR2s open, the distance to membrane, the distance to the closest spark, the amplitude, the time to reach the peak, the full duration at high maximum (FDHM), the number of RyR2s that fire and the number of CaRUs that fire. These results show that, in rest conditions, the spark properties do not change with the cell t-tubule density.

References

[1] D. M. Bers. Cardiac excitation–contraction coupling. *Nature*, 415(6868):198, 2002.

[2] M. Marchena and B. Echebarria. Computational model of calcium signaling in cardiac atrial cells at the submicron scale. *Frontiers in Physiology*, 9:1760, 2018.

Table 1: Parameters.

**General parameters**		
time step	Δt (ms)	
spatial step	Δx (μm)	0.1
Resting potential	V_{res} (mV)	-85
Maximum potential	V_{max} (mV)	10
cell x-length	L_x (μm)	100
cell y-length	L_y (μm)	15
volume factor (cytoplasm)	v_i/v_{sr}	200
volume factor (SR)	v_i/v_{sr}	20
volume myocyte	v_{myo} (μm^3)	21500
Temperature	T(K)	308
Faraday constant	F(C/mol)	96485
Ideal gas constant	R(J/(Kmol))	8.31
**Buffer Parameters**		
TnC concentration	B_{TnC}(μM)	70
TnC binding rate	$k_{on,TnC}$(μM^{-1}ms^{-1})	0.00327
TnC unbinding rate	$k_{off,TnC}$(μM^{-1})	0.00196
Calmodulin concentration	B_{CaM}(μM)	24
Calmodulin binding rate	$k_{on,CaM}$(μM^{-1}ms^{-1})	0.003
Calmodulin unbinding rate	$k_{off,CaM}$(μM^{-1})	0.02
SR-bound buffer concentration	B_{SR}(μM)	23.5
SR-bound buffer binding rate	$k_{on,SR}$(μM^{-1}ms^{-1})	0.01
SR-bound buffer unbinding rate	$k_{off,SR}$(μM^{-1})	0.006
Calsequestrin buffer concentration	B_{CSQ}(μM)	2400
Calsequestrin buffer dissociation constant	$k_{d,CSQ}$(μM)	2000
**Na-Ca exchanger**		
Uptake strength exchanger	g_{NaCa}(μM/ms)	172
Extra cellular Ca concentration	$[Ca]_o$(mM)	1.8
Extra cellular Na concentration	$[Na]_o$(mM)	136
Intra cellular Na concentration	$[Na]_i$(mM)	10
Inactivation constant	K_{da}(μM)	0.275
Saturation constant	k_{sat}	0.27
Voltage sensitivity constant	η	0.35
External sensitivity constant for Na	K_{mNao} (mM)	87.5
External sensitivity constant for Ca	K_{mCao} (mM)	1.3
Internal sensitivity constant for Na	K_{mNai} (mM)	12.3
Internal sensitivity constant for Ca	K_{mCai} (mM)	0.0036
**SERCA parameters**		
Maximum uptake SERCA	g_{up}(μMms^{-1})	0.09
Half occupation of cytosolic calcium binding states	K_i(μM)	0.0615
Half occupation of SR calcium binding states	K_{sr}(mM)	1.3

Table 2: Parameters.

**General parameters**		
**RyR2 parameters**		
Single channel strength	$g_{rel}(\text{ms}^{-1})$	4
Number of RyR2 per CaRU	N_{RyR2}	4-9
Closing rate	$k_{oc} = k_{i1i2}(\text{ms}^{-1})$	0.08
Inactivation rate	$k_{oi} = k_{ci}(\text{ms}^{-1})$	0.001
Recovery rate from $I \to C$	$k_{ic}(\text{ms}^{-1})$	0.0025
Opening rate parameter	$k_a(\mu\text{M}^{-2}\text{ms}^{-1})$	$2.1 \cdot 10^{-3}$
Transition in I states	$k_b(\mu\text{M}^{-2}\text{ms}^{-1})$	10^{-4}
Maximum value of k_{CaSR}	Max_{SR}	15
Minimum value of k_{CaSR}	Min_{SR}	1
Exponent in SR calcium lumenal dependence	H	5
Threshold concentration of SR calcium	$EC_{50-SR}(\mu\text{M})$	450
**LCC parameters**		
Strength effective flux	g_{CaL} $(\mu m^3/\text{ms})$	32610
Number of LCC per CaRU	N_{LCC}	5
Threshold Ca-induced transition	$K_{LCC}(\mu\text{M})$	5

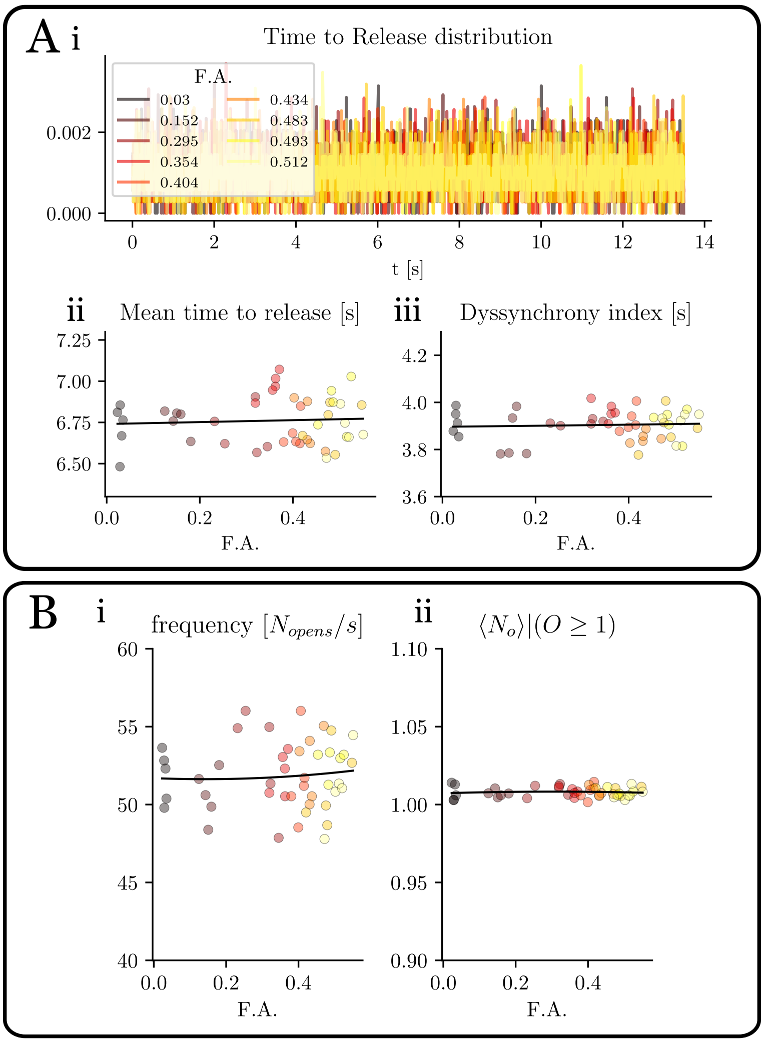

Figure 3: Simulations without external potential. A: time to release distribution (i), average time to release (ii) and dyssynchrony index (iii) as function of the F.A. B: Dependence on the F.A. of (i) number of RyR2s that open per second and (ii) average number of openings for those RyR2s that have opened at least once. Total time of simulation: 13.5s.

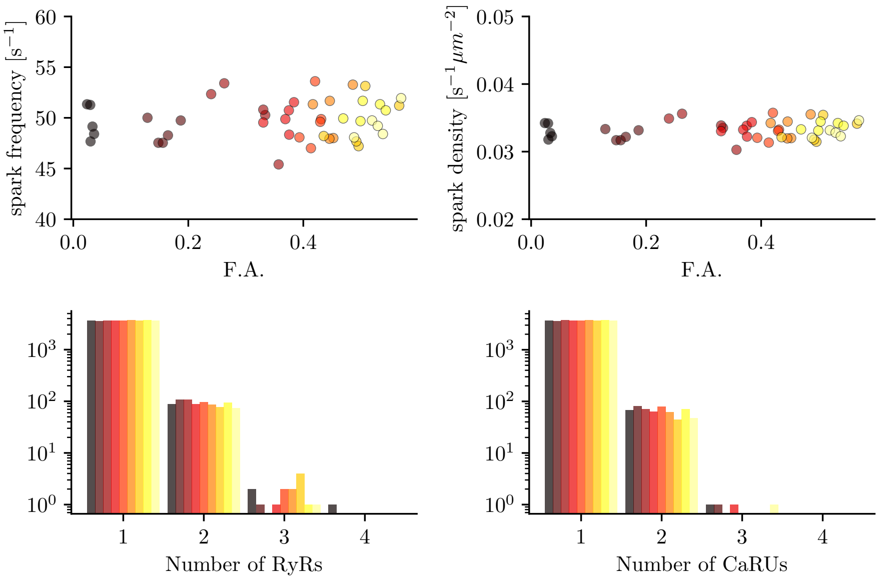

Figure 4: Spark frequency (i) and density (ii) as function of F.A. Number of RyR2s (ii) and CaRUs (iv) involved in each event.

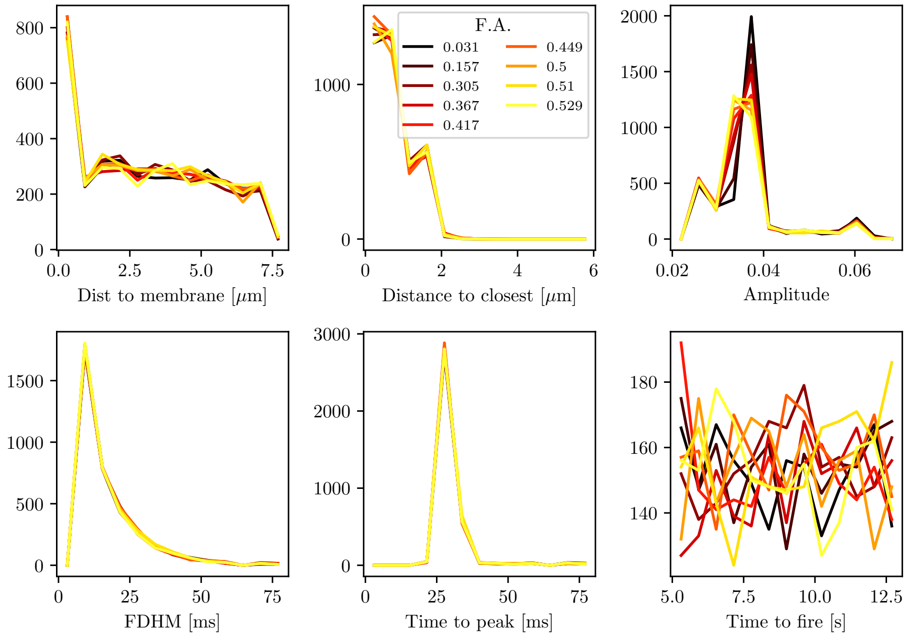

Figure 5: Histograms corresponding to spark properties for different values of the fractional area.
